# Alternative, Complementary, and Forgotten Remedies for Atopic Dermatitis

**DOI:** 10.1155/2015/676897

**Published:** 2015-07-15

**Authors:** Allison L. Goddard, Peter A. Lio

**Affiliations:** ^1^Brigham Dermatology Associates, 221 Longwood Avenue, Boston, MA 02115, USA; ^2^Medical Dermatology Associates of Chicago, 363 W. Erie Street, Suite 350, Chicago, IL 60654, USA

## Abstract

Atopic dermatitis, perhaps more than other dermatologic diseases, has garnered much attention in the realm of alternative medicine. This may be because its etiopathogenesis is incompletely understood, it is increasingly common, and it waxes and wanes often without clear precipitants, opening up many opportunities for misinterpretation. Herein we explore the evidence for a number of different alternative and complementary therapies, from textiles to vitamin supplements. By definition, none have enough data to be deemed “effective” in a conventional sense, but it is hopeful that some show promising evidence that may one day lead to mainstream acceptance with further research.

## 1. Introduction

Atopic dermatitis (AD) is a chronic and intermittent inflammatory dermatosis, which carries with it significant morbidity. There seems to be tremendous interest in alternative and complementary approaches to treating AD perhaps because it sits in the eye of a perfect storm of attributes: its etiopathogenesis remains obscure, it is increasingly common, and it waxes and wanes often without clear precipitants, opening up many opportunities for misinterpretation.

A functional definition for alternative and complementary medicine can simply be approaches that are not evidence-based. Overwhelmingly, however, this is because there is simply* insufficient* evidence rather than evidence* against* the therapy. In what follows, we will delve into the literature for a number of different alternative and complementary approaches, focusing on the existing evidence. Sometimes this will be more compelling than others, but our hope is that it is always informative, at the very least, and may help guide discussions with patients who request—or even insist on—alternative approaches.

## 2. Textiles

Wool intolerance is a well known feature of AD [1]. Wearing alternative fabrics such as cotton and silk has been shown to reduce pruritus and aid in emollient absorption [2, 3]. A number of sophisticated “functional textiles” have been developed with the intention of aiding in the management of atopic dermatitis. The use of fabrics impregnated with antimicrobial materials such as silver, zinc, and “anions,” as well as specially coated antimicrobial silk, has been investigated in AD with some benefit [4–9]. Fabrics such as borage oil-treated garments have also been developed to improve skin moisture and restore lipids [10]. Lopes et al. recently performed a meta-analysis of 13 studies evaluating these specialty textiles and their reported impact on measures such as SCORAD index, symptom reduction, need for rescue medication, quality of life measures, skin colonization of* Staphylococcus aureus*, transepidermal water loss, and overall safety [11]. This study found evidence for recommendation of the use of functional textiles in AD to be weak although generally safe. Cost of these specialized garments is another consideration. For these reasons, further investigation into the efficacy of textiles is warranted before recommending strongly them to our patients.

## 3. Climate and Temperature

Physicians who treat patients with AD are aware that the physical environment plays an important role in their patients' lives. Regional variation in incidence of AD within the United States, as well as globally, supports the idea that climate factors impact this disease. In 2013, a large-scale ecological examination of the relationship between eczema prevalence and the physical environment within the United States was published. In this large, population-based study there was reduced eczema prevalence in areas with high relative humidity, high UV index, high mean temperature, reduced precipitation, and fewer days of central heating use [12].

Climatotherapy is an alternative treatment option that has been utilized for many years for both atopic dermatitis and asthma. It involves specialized eczema clinics where patients stay for a period of weeks to months that combines anti-inflammatory therapies with a “healing” environment. Both seaside and mountain clinic resorts exist. A recent systematic review of the literature on climate therapy included 11 studies with over 39,000 patients with AD who were treated in centers in Davos, Switzerland, elevation 1560 meters, or in various centers in Germany at elevations ranging from 1000 to 1600 meters above sea level. Disease activity was decreased in the majority of patients at the end of climatotherapy based on Physicians Global Assessment (four studies, 39,052 patients) or eczema severity indices such as SCORing Atopic Dermatitis (SCORAD) (four studies, 451 patients) [13]. Topical and systemic corticosteroid use was also reduced. Although follow-up data was not well documented in all studies, four studies with 2670 patients collectively reported that, at 12 months of follow-up, 64% of patients had continued decrease in disease activity compared with the year prior to therapy [13]. In addition, the majority reported reduced or no topical steroid use at 12 months of follow-up [13]. Proposed mechanisms of climatotherapy include a lack of or low burden of respiratory allergens at higher elevation, as well as atmospheric pressure and air temperature optimization for balance of heat and water loss in the skin [13, 14]. These climate therapy programs are not practical for most patients, however, but may be something to consider in a select patient population and support observations regarding the impact of climate on patients with AD.

A single study looked at whole body cryotherapy using a specialized chamber at temperatures as low as −110°C (−166°F) three times weekly [15]. The authors demonstrated improvement in patients with severe AD and hypothesized that the beneficial effects were related to reduced nerve conduction velocity and decreased acetylcholine synthesis, thereby influencing pruritus. Although this concept is interesting, the practicality of using this treatment chamber in routine clinical practice is not realistic. We know from clinical experience that the use of short-contact ice packs can be helpful in stopping localized pruritus in a number of dermatologic conditions and may be analogous.

From a physiologic standpoint, it is well-established that the stratum corneum and barrier function in AD is compromised [16]. An important role of the stratum corneum is to provide homeostatic mechanisms to protect and adapt to environmental changes such as humidity, temperature variability, and UV exposure. When this barrier is impaired, physical and environmental stressors can be detrimental. Future research and further understanding of the association between eczema and climatic factors are needed and could potentially lead to climate-specific therapeutic interventions.

## 4. Water and Bathing

Therapeutic bathing, or balneotherapy, involves immersion in mineral water baths or pools, often in the context of warm weather (climatotherapy; see above) and sun exposure (heliotherapy) and in a relaxing or soothing environment and has shown some promise in evidence-based therapeutic trials.

The healing powers of the Dead Sea in Israel have attracted patients with a wide variety of ailments for centuries. The waters are rich in minerals believed to promote skin healing, particularly in combination with sun exposure. A study of 49 AD patients that involved bathing in the Dead Sea for 20 minutes, twice daily, with subsequent increasing gradation of sun exposure, found significant improvement in eczema severity and quality of life measures [17]. Similar studies have shown benefit in psoriasis and vitiligo [18].

Another natural mineral spring with potential healing properties exists in the South of France. A 3-week course of daily bathing in the Avène hydrotherapy spa led to sustained improvement in quality of life measures in both AD and psoriasis patients after three and six months of follow-up [19]. Proposed mechanisms for the benefit of these natural minerals include modulation of IL-8, inhibition of TNF-alpha-induced adhesion molecules, and reduced staphylococcal skin colonization [20, 21]. Salt water with added minerals may also modulate Langerhans cells and inhibit 5-lipoxygenase and Th1 lymphocytes [22].

Extended vacations to Israel or the South of France are not possible for most patients; therefore several attempts have been made to replicate these natural, mineral-rich spring water conditions. A prospective randomized study of 180 AD patients examined balneophototherapy, which involved bathing in a 10% Dead Sea salt solution plus narrow band ultraviolet B (UVB) light, compared to standard narrow band UVB phototherapy alone. This study demonstrated statistically significant superiority in the combined therapy group (with a 26% difference in improvement between groups, *P* < 0.001) on the SCORAD after 35 sessions [23].

Interestingly, in contrast to the proposed benefits of natural mineral waters in inflammatory skin disease, epidemiological studies exist which suggest that hard water (another term for water high in mineral content) may worsen atopic dermatitis. This propelled a study examining the benefits of commercial water softeners. This large randomized trial of 336 children found no difference in patients randomized to use of a water softener versus those bathing in hard water systems [24]. Overall, it appears that mineral baths alone may not be sufficient to improve eczema; however, the beneficial effects appear to be substantially augmented when phototherapy is used concurrently.

## 5. Dilute Bleach Baths

Salt minerals are not the only healing products that can be added to the bath water of eczema patients.* Staphylococcus aureus* colonization has been linked to worsening of disease activity and is the most common infectious complication seen in AD [25]. In 2009, a landmark study, by Huang et al., demonstrated significant improvement in eczema severity with use of dilute bleach baths [26]. The study compared an experimental group receiving dilute bleach baths (0.005% final concentration, approximately 0.5 cup of 6% sodium hypochlorite in a full bath) for 5–10 minutes twice weekly, combined with intranasal mupirocin ointment, compared to a placebo-bleach bath and intranasal petrolatum control group [26]. There were statistically significant reductions in eczema area and severity index scores, at one and three months, compared to placebo group. Recommending dilute bleach baths has since become mainstream for many patients with moderate-to-severe AD, particularly those with frequent secondary infections.

A more recent study evaluated a sodium hypochlorite “bleach cleanser” in a 12-week, open-label protocol for 18 children with AD [27]. The study did not have a control group, but the treated patients did show clinical improvement by Investigator Global Assessment (IGA) eczema score and decrease in body surface area of affected skin. Further studies to validate this effect are warranted.

## 6. Moisturization

Epidermal barrier failure is thought to be a main driving force behind subsequent proinflammatory cascades in AD and skin moisturization is a fundamental aspect in controlling flares. Emollient use alone has shown to have significant effects on eczema severity and quality of life [28]. Regular emollient use has been demonstrated to have a steroid-sparing benefit, which is a paramount goal in managing atopic dermatitis [29].

The critical question remains: Is there a “best” emollient? The answer is not simple and is becoming more complex as progressively more innovative formulations arise in our armamentarium of product options. As we learn more about the causes of xerosis and barrier dysfunction in AD, companies strive to develop products aimed at targeting these deficiencies. These products frequently claim to address at least one of the three leading causes of xerosis in AD: deficiency of natural moisturizing factor (NMF); deficiencies in ceramides; and deficiency in aquaporin water channels [30].

The vast array of drugstore moisturizers can be overwhelming for patients and practitioners alike. As the products on the shelves claim more specialized formulas targeted at specific issues in atopic skin, pharmaceutical companies have also produced a number of prescription “barrier repair creams.” Although a wide variety of these creams exist, many contain one or more of the following ingredients, each of which has measurable effects on skin barrier function and inflammatory mediators: ceramides, hyaluronic acid, dimethicone, licorice extract (glycyrrhetinic acid), and palmitoylethanolamide. Many of these ingredients can also be found in over-the-counter (OTC) products, although in varying concentrations and combinations. “Barrier repair creams” are available in the USA as prescription 510 (k) devices (a markedly different category than true drugs) yet tend to be priced as much as or more than topical drugs, often in the hundreds of dollars range [31]. A randomized controlled trial looked at the cost-benefit ratio between two leading barrier creams (one containing licorice extract, the other containing ceramides) and OTC petroleum based moisturizer. This study found the OTC product not only to be more effective, but also, more importantly, to be 47 times more cost-effective than the prescription barrier creams [32].

Moisturizers are a daily part of skin care in atopic dermatitis and patients often use a variety of products based on personal preferences, skin type, season, and disease state. Similar to sunscreen recommendations, personal preference is critical, and patients will not use what they do not like. Hence it is crucial to consider patient preference and subsequent adherence, rather than approaching emollient recommendations as a “one-size-fits-all” process.

## 7. Natural Oils

Sunflower seed oil (*Helianthus annuus*) has been shown to have both anti-inflammatory and barrier restoring effects [33]. Its major lipid is linoleic acid, which is thought to decrease inflammation in the skin by activating peroxisome proliferative-activated receptor-alpha (PPAR-*α*). Sunflower seed oil, particularly in its oleodistillate form (SOD), is believed to improve the skin barrier [33]. Sunflower seed oil (SSO) was found to be comparable to Aquaphor in reducing infant mortality and rates of sepsis (26% reduction in preterm neonate mortality for SSO, 32% reduction for Aquaphor) by enhancing skin barrier recovery [34]. A follow-up study found both interventions to be effective in terms of reducing infant death rate; however, SSO was felt to be superior in terms of cost [35]. These studies can be applicable to our AD population in that compromised skin barrier is a universal issue.

Another small RCT looked at the effect on skin barrier of olive oil compared to SSO in 19 adults both with and without atopic dermatitis. Olive oil was found to be detrimental to the skin barrier, while SSO preserved stratum corneum integrity, did not cause the erythema that the olive oil did, and improved skin hydration [36]. Virgin coconut oil (VCO,* Cocos nucifera*) has shown benefit as both an excellent emollient and natural antibacterial against* S. aureus*, in addition to demonstrating anti-inflammatory activity in animal models of both acute and chronic inflammation [37, 38]. Virgin coconut oil is defined by the method in which it is processed, allowing VCO to retain its natural antioxidant and antimicrobial properties. A recent randomized double-blind trial in 117 pediatric AD patients looked at the effects of VCO compared to mineral oil on SCORAD index values, transepidermal water loss (TEWL), and skin capacitance in 117 pediatric AD patients over an eight-week period. Mean SCORAD indices decreased from baseline by 68.23% in the VCO group versus 38.13% in the mineral oil group (*P* < 0.001) and overall VCO was found to be superior to mineral oil in all objectives [39].

## 8. Wet-Wraps

Wet-wraps take moisturization to another level and can produce rapid therapeutic benefit for patients. The process involves applying topical medication and/or moisturizer to damp skin, followed by a layer of damp cloth or gauze, and subsequent application of a dry layer of cloth or gauze. These layers are left in place for several hours or overnight while sleeping. Occlusive moisturization increases water content in the skin and improves the epidermal barrier [40]. The physical barrier of the wrap can also reduce trauma associated with scratching, particularly overnight.

A review of the literature including 24 publications on wet-wrap therapy noted that despite variation in study methodology and wet-wrap technique, this practice is effective for moderate-to-severe AD in the short term. The review concluded that use of topical corticosteroid as a component of the wet-wrap was more effective than emollients alone. The use of topical steroids with wet-wrapping not only appears to be safe for up to 14 days, but also decreases the total amount of corticosteroid used during that period compared to those without wet-wrapping. Discomfort was the main adverse event reported [41].

Occlusion, whether wet or dry, does increase the density of bacteria on the skin, and a common complication of wet-wrap therapy was folliculitis. Although decolonization of skin bacteria with dilute bleach baths prior to wet-wrap application has not been evaluated, it theoretically may decrease incidence of folliculitis and is a hypothesis worthy of exploration. Similarly the use of nut oils that contain inherent antimicrobial properties, such as coconut oil, as the emollient of choice in the wet-wrap, has not been investigated.

## 9. Allergan Avoidance/Avoidance of Triggers

The concept of atopy and its role in atopic dermatitis has been questioned by some experts, as not all patients with AD have allergic triggers to disease flares [42]. For those that do, there is some evidence to support that airborne proteins, such as those produced by house dust mites (HDM) or cockroaches, have proteolytic activity that may affect skin barrier function and drive immunologic inflammation in AD patients [43]. There was a period of time in which recommending special pillow and mattress covers to AD patients in order to reduce HDM allergen exposure was quite common amongst dermatologists. This has fallen out of favor because of lack of observed benefit. It is also notable that the largest and most promising paper supporting this recommendation was deeply flawed [44].

For some patients, identifying allergen and irritant triggers can be beneficial in the control of their disease, but there is no sufficient data to support implementing allergen control measures universally.

## 10. Immunotherapy

Beyond mere avoidance of allergens, desensitization to certain allergic triggers may be achieved with immunotherapy. Allergen-specific immunotherapies such as those for HDM have shown to be helpful in asthma and allergic rhinitis. A number of AD patients also have sensitization to HDM although the association between HDM and eczema remains controversial [45]. Overall data is lacking to make a strong recommendation for the routine use of immunotherapy in AD at this time, but preliminary studies yielded beneficial results in HDM-sensitized patients, particularly in those with milder cases of AD. Ideally, further studies will continue to investigate this specific therapy and its impact on patients with AD [46].

## 11. Diet

The exact influence of food exposure on AD remains unclear, although it is a frequent suspect for the “root cause” of eczema amongst many families and some healthcare practitioners. One-third of moderate-to-severe AD patients have verifiable, type I hypersensitivity food allergies, resulting in rapid onset urticaria, angioedema, or anaphylaxis [47]. These immediate type I hypersensitivity IgE reactions, however, are felt to be separate compared to eczematous reactions or flares of AD, which would be delayed 6–48 hours after exposure.

Food allergy testing traditionally involves skin prick testing (SPT) and serum-specific IgE levels, which assess for type I hypersensitivity reactions. Negative tests help to rule out allergy; however, positive tests only indicate sensitization and are not felt to be helpful in predicting skin reactions; therefore food allergy testing is generally not recommended for patients with symptoms limited to eczematous skin lesions [48, 49]. Because of this disconnection, investigators have looked at atopy patch tests (APT: applying patch test preparations of foods directly to the skin for 24–48 hours) to assess for type IV hypersensitivity, which more commonly yields eczematous type skin reactions. Studies examining the utility of APT in relation to patient-specific atopic dermatitis flares have been conflicting and therefore are also not routinely recommended [50, 51].

Thompson and Hanifin demonstrated that approximately 80% of perceived food associations, as the underlying cause for atopic dermatitis flares (rather than triggers for type I hypersensitivity responses), disappear once the skin disease is under better control [52]. An excellent review in 2009 very nicely summarized the current literature on dietary exclusions in AD. This review concluded that there is some evidence to support avoidance of eggs in infants with specific serum IgE antibodies to eggs. The authors found no benefit to exclusion of foods in patients with uncertain IgE status. Finally there may be small contributions of certain foods, such as gluten, causing nonallergy driven inflammation in some patients, leading to worsening of AD; however such associations are not yet clearly defined [53].

## 12. Gluten Avoidance

Outside of celiac sprue and the associated skin condition dermatitis herpetiformis, the concept of gluten sensitivity and its proposed associations with inflammatory skin disease has become commonplace enough to warrant expanded gluten-free sections of supermarkets. Well before this widespread discussion, in 1985 one small study found that 30% of adults with atopic eczema had detectable serum IgG antibodies to gliadin (a component of gluten), compared to only 6.5% of the general population [54]. In 2004, another study looked at over 1,000 patients with celiac disease and found that atopic dermatitis was about three times more common in these patients than in the general population [55]. Unfortunately, however, gluten avoidance for one year did not improve flares of atopic dermatitis in these patients. An Italian group looked at serum of 401 atopic dermatitis patients and found that four patients (1%) had detectable antibodies to both IgA antiendomysial and IgA anti-tissue transglutaminase. Eight patients had anti-gliadin IgG antibodies. None had clinical evidence of malabsorption or gastrointestinal symptoms, but all four were confirmed to have celiac by duodenal biopsy. The patients were placed on a gluten-free diet, but the study was published before they were able to assess any impact on their eczema [56].

Although there is very scarce data to support it, it may not be unreasonable to support healthy adults who wish to try gluten avoidance for several months, particularly in difficult cases when other interventions have failed.

## 13. Formula

For infants with AD, hydrolyzed formulas have been developed with atopic dermatitis and allergic disease in mind. Hydrolyzed or partially hydrolyzed formulas have broken-down, more easily digestible whey proteins. Amino acid-based formulas are available for infants intolerant to cow's milk formula. Researchers have looked at prevention of AD with various hydrolyzed formulas when breast feeding was not an option. A 6-year follow-up study of a group of 2,252 at-risk newborns, who were randomized to various hydrolyzed formulas, found significant risk reduction for allergic disease in those receiving hydrolyzed or partially hydrolyzed formulas as infants when compared to those who received cow's milk formula [57]. However, it is difficult to know whether or not there is an effect for AD alone. Given the expense, without sufficient evidence, switching formulas cannot be recommended in the absence of other allergic or intolerance symptoms.

## 14. Supplementation

Although the benefits of vitamin D in AD remain uncertain, several small studies offer promising results. A group of children with AD in Boston, who all reported worsening of their eczema in the winter, were randomized in a small pilot study to receive vitamin D supplementation or placebo for one month during the winter. The results demonstrated significant improvement in eczema in 80% of the treatment group compared to 17% of placebo group [58]. This benefit was later confirmed with a larger randomized double-blind placebo-controlled trial of 107 AD children during the winter in Ulaanbaatar, Mongolia [59]. Another small study demonstrated increased benefit from combining vitamin D with vitamin E supplementation compared to either vitamin supplementation alone [60]. Interestingly, topical vitamin D (and its analogs), while helpful in psoriasis, may actually flare AD via triggering of Th2 inflammation [61].

Despite theoretical potential benefit, no clinically significant benefit has been seen in supplementing pyridoxine (vitamin B6), zinc phosphate, selenium, sea buckthorn seed, or hempseed oil, although current data is sparse [56, 62, 63].

Topical B12 (cobalamin) has been shown to successfully improve atopic dermatitis in both children and adults [64]. The proposed mechanism is nitric oxide synthase inhibition, an important step in one AD inflammation pathway. Larger studies are needed to support these early encouraging findings [64, 65].

## 15. Fatty Acids: Evening Primrose, Borage Oil

Gamma linoleic acid (GLA), a polyunsaturated fatty acid, is a precursor of inflammatory mediators such as prostaglandin E and is the primary substrate for arachidonic acid [66]. It has been suggested that the enzyme responsible for converting linoleic acid to GLA (delta-6-desaturase) may be deficient in in patients with AD [67]. Evening primrose oil (*Oenothera biennis*, EPO), black currant seed oil (BCSO), and borage oil (BO) contain relatively high levels of GLA and therefore have been an area of investigation in the realm of supplemental management in AD.

A small RCT evaluated EPO supplementation (2,000–6000 mg daily) in children and adults with AD and reported reduced itching and intensity of symptoms when compared to placebo [68]. In another RCT, 313 pregnant mothers with strong atopic history were supplemented with BCSO or olive oil as placebo starting at 8–16 weeks' gestation and continuing throughout the duration of breastfeeding. Infants born to these mothers in the BSCO group were found to develop lower than expected prevalence of AD in the BSCO group at 12 months of age as well as lower SCORAD indices in those infants affected. However, no differences were seen between groups at 24 months [69]. A 2013 Cochrane review, assessing the effects of EPO or BO on treating symptoms of AD, failed to show benefit from either oral fatty acid supplement when compared to placebo. The analysis included 27 studies and nearly 1600 participants [70].

## 16. Probiotics

The interest in prebiotics (nondigestible oligosaccharides that support microbiota) and probiotics is expanding for a number of inflammatory diseases, including atopic dermatitis. Over the years, multiple, well designed trials have noted benefit in improving the severity of AD or potentially preventing onset of AD in high-risk neonates. The strains of probiotics investigated as oral supplements include* Lactobacillus* species such as* L. rhamnosus*,* L. plantarum*,* L. salivarius*, and* L. acidophilus*, as well as* Bifidobacterium breve*. These strains have been studied in various combinations and as monotherapy.

Recent preliminary data looking at* L. salivarius* LS01 in 43 children, aged 0 to 11 years, reported significant improvement in SCORAD indices and pruritus in all subjects after four weeks. Final, longer term results are pending [71].

RCT from 2001 demonstrated that* L. rhamnosus* GG had preventative effects and reduced development of AD in 132 at-risk infants through the age of seven years [72]. A recent meta-analysis review of 25 RCTs for the management of AD in different age groups concluded that improvements in SCORAD values were observed in the probiotics group over the control group in both children and adults. The analysis, however, could not extract supporting evidence for a beneficial role of probiotics in infants less than one year old. The authors did not note a significant benefit with symbiotic use (prebiotics combined with probiotics) over probiotics alone [73]. Many questions remain, including which organism or combination of organisms is most effective for AD, what is the proper dosing and duration of treatment, and which subset of patients would potentially benefit the most.

Topical probiotic extracts are in the phases of early development and will ideally show activity against pathogenic biofilms. Additional data is needed to support this preliminary work.

## 17. TCM and Acupuncture, Herbal Medicine

A Cochrane review in 2004 examined the collective evidence for Chinese herbal medicine (CHM) in atopic dermatitis. Because most studies were small, they could not make strong recommendations but did see a trend in positive benefit [74]. Another meta-analysis in 2013, by Tan et al., included seven RCTs comparing either CHM with western medicine (WM) versus WM alone or CHM versus placebo. Results of the meta-analyses revealed significant improvement in disease severity scores, improvement in erythema, pruritus, sleep scores, and quality of life, and need for concurrent pharmacotherapy. Studies reported that CHM was well tolerated with no serious adverse events, although reversible transaminitis was mentioned in at least one study. Unfortunately, even with the unanimously positive benefits reported with CHM, the authors were still unable to reach valid conclusion of true statistical benefit due to poor study design and heterogeneity of the protocols. The formulas used for AD varied between each clinical trial and this correlates with the lack of standard formulation in CHM clinical practice. The exact pharmacokinetic and pharmacodynamics of individual herbs differ greatly and are often poorly understood, particularly when taken in varied combinations [75]. In addition, herbs can be contaminated with heavy metals or even steroids, further confusing the picture. Of note, the formulas used in the RCT reviewed by Tan were screened for contaminants.

Acupoint stimulation has been successful in reducing pruritus associated with a number of pruritic conditions, including atopic dermatitis. Previous studies have shown that Quchi (LI11) is the acupoint most commonly used for pruritus management [76]. Lee et al. conducted a randomized trial using self-administered acupressure applied at the Quchi point and found decreased pruritus and lichenification after four weeks [77]. Acupuncture may also modulate histamine mediated itch and allergic rhinitis [78]. Although histamine does not equal eczema, it may be one of many triggers.

## 18. Homeopathy

Homeopathy was developed by Samuel Hahnemann in 1796 and, in simple terms, uses extremely dilute preparations (some so dilute that not a single molecule of the agent remains) to treat a variety of ailments. There are no quality RCTs looking at homeopathy for AD. One reason for the lack of controlled studies is that the very nature of homeopathy is based on and individualized approach to patient's precise symptoms following a detailed consultation. Clinical homeopathy is a subtype of homeopathy that is typically tailored to a specific disease entity rather than to a more in-depth personalized prescription. To date, there has been no convincing evidence for a therapeutic effect of homeopathy in AD. In addition, any positive effects seen in other dermatologic diseases have not been widely reproducible [79]. Despite this, we know that the placebo effect has been found to have measurable physiologic improvement in disease and may contribute to benefit in homeopathy in some patients [80].

## 19. Psychological and Educational Interventions

Stress can negatively impact the immune system via a number of proposed mechanisms [81]. Massage, relaxation, biofeedback, hypnosis, and aromatherapy have been explored as complimentary approaches in treatment of AD. The majority of psychological and educational intervention studies use these techniques as adjuvant modalities in combination with conventional topical therapies.

Coping with stress through support groups and progressive muscle relaxation has shown to have a positive influence on patients' quality of life, pruritus scores, and loss of sleep, but there is less evidence that it has an effect on eczema severity indices [82, 83]. Biofeedback and hypnosis were combined in one study and compared to a control group. In this study, the intervention group demonstrated significant reduction in an eczema severity score at 8 and 20 weeks [84].

Multidisciplinary structured educational programs for children with AD and their families have shown benefit in reducing eczema severity and improving quality of life measures [85]. A recent Cochrane review evaluated 10 studies using educational interventions and psychological interventions as adjunct therapies to conventional treatment for children with AD. Although data was not sufficient for a comparable meta-analysis, the authors report that there is evidence to demonstrate benefit from structured educational and psychological interventions in terms of eczema severity and quality life for both children and families [86].

The role of education must begin in the exam room with the patient. Studies have demonstrated that patients recall between 40 and 80% of the information they receive during an office visit [87, 88]. Treatment regimens can be complex, particularly for patients with moderate-to-severe disease. Recall is improved with the combination of verbal and written instruction. Shi et al. looked at 37 patients, randomized to receive either verbal instruction alone or the verbal instruction plus a customized written eczema action plan at the end of the clinic visit. The addition of the written action plan only added 3–5 minutes to the visit but resulted in significant improvement in patients understanding of their individualized daily treatment plan, benefits and risks of the prescribed medication use, and duration of treatment, recognizing AD exacerbating factors, comfort in adjusting treatment based on disease severity, and reduced anxiety for AD care at home [89].

## 20. Conclusion

In addition to the four main pillars of conventional AD treatment, moisturization, anti-inflammatories, antibacterials, and antipruritics [90], there is a universe of other approaches that can be used as alternatives to or complementary with those therapies. While these are largely unproven, many have compelling scientific rationales and promising preliminary evidence to warrant further study. As the incidence of AD rises, one thing is certain: one size will certainly not fit all. Having a large palette of potential treatments allows for better customization for patients and provides a resource for continued hope in the face of an incurable and often demoralizing disease.
